# Activation of p38MAPK Contributes to Expanded Polyglutamine-Induced Cytotoxicity

**DOI:** 10.1371/journal.pone.0002130

**Published:** 2008-05-07

**Authors:** Maria Tsirigotis, R. Mitchell Baldwin, Matthew Y. Tang, Ian A. J. Lorimer, Douglas A. Gray

**Affiliations:** 1 Centre for Cancer Therapeutics, Ottawa Health Research Institute, Ottawa, Ontario, Canada; 2 Department of Biochemistry, Microbiology and Immunology, University of Ottawa, Ottawa, Ontario, Canada; University of Cambridge, United Kingdom

## Abstract

**Background:**

The signaling pathways that may modulate the pathogenesis of diseases induced by expanded polyglutamine proteins are not well understood.

**Methodologies/Principal Findings:**

Herein we demonstrate that expanded polyglutamine protein cytotoxicity is mediated primarily through activation of p38MAPK and that the atypical PKC iota (PKCι) enzyme antagonizes polyglutamine-induced cell death through induction of the ERK signaling pathway. We show that pharmacological blockade of p38MAPK rescues cells from polyglutamine-induced cell death whereas inhibition of ERK recapitulates the sensitivity observed in cells depleted of PKCι by RNA interference. We provide evidence that two unrelated proteins with expanded polyglutamine repeats induce p38MAPK in cultured cells, and demonstrate induction of p38MAPK in an *in vivo* model of neurodegeneration (spinocerebellar ataxia 1, or SCA-1).

**Conclusions/Significance:**

Taken together, our data implicate activated p38MAPK in disease progression and suggest that its inhibition may represent a rational strategy for therapeutic intervention in the polyglutamine disorders.

## Introduction

The polyglutamine diseases encompass at least 9 different disorders including Huntington's disease (HD) and five spinocerebellar ataxias (SCA-1, SCA-2, SCA-3, SCA-6 and SCA-7 (reviewed in [1]). These are dominantly inherited diseases typically detected in the third or fourth decade of life. No effective therapeutic interventions are currently available, and the polyglutamine diseases are generally fatal. Polyglutamine disorders arise from expansion of a CAG repeat within the coding region of genes such that the length of the encoded polyglutamine stretch exceeds a critical threshold. At the ultrastructural level, disease progression features heat shock protein (HSP)-containing nuclear ubiquitinated inclusions [2] that have accumulated an assortment of cellular host components in association with the polyglutamine-containing protein [3]. There is evidence from experiments performed in cultured mammalian cells and animal models of disease that polyglutamine expanded proteins adversely affect basic biological processes (reviewed in [4]). Their expression has been associated with impaired proteolysis [5], loss of transcriptional control mechanisms [6] and with altered regulation of cell death/survival pathways (reviewed in [7]).

The mitogen-activated protein kinases (MAPK) are involved in the integration and processing of multiple extracellular signals and their induction triggers diverse biological responses (reviewed in [8], [9]). While the activation of the extracellular regulated kinase 1/2 (hereafter referred to as ERK) by mitogenic and proliferative stimuli is coupled to cell survival [10], stress inducible kinases JNK and p38MAPK respond to environmental stress and their sustained activation transduces signals leading to cell death (reviewed in [11]). Protein kinase C (PKCs) family members have been positioned upstream of ERK and are potent modulators of its activation (reviewed in [12]). With the current exception of the stress-inducible kinase JNK whose excessive activation has been well documented in neurodegenerative diseases [13] and reviewed in [14], the mechanistic relationship between the stress inducible host signaling pathways and expanded polyglutamine-induced toxicity remain controversial. It has been shown, for example, that the mutant huntingtin (Htt) protein causes aberrant activation of epidermal growth factor receptor (EGFR) signaling [15], a finding which has been contradicted by more recent reports in which EGFR signaling was disrupted by expression of the expanded polyglutamine protein [16], [17]. In a *Drosophila* model of polyglutamine toxicity, the mutant Htt protein has been shown to disrupt EGFR signaling through interference with the ERK cascade [18] while in a cell culture model it has been shown to activate the pro-survival pathway mediated through ERK [19]. All these anomalies are consistent with gain of function effects of expanded polyglutamine proteins. There is ample evidence from experimental systems that a simple polyglutamine tract can be toxic without the context of its natural surrounding protein sequence [20], [21] but possible loss of function effects in polyglutamine proteins must also be considered. The normal huntingtin protein, for example, has been shown to increase transcription of brain-derived neurotrophic factor (BDNF), which is required for survival of striatal neurons [22], [23]. Loss of this activity in the mutant protein may therefore contribute to neuronal loss in diseased individuals. Insulin-like growth factor I also has neuroprotective activity in the context of polyglutamine-induced cytotoxicity [24], [25], and like BDNF activates the survival pathway mediated through the phosphoinositide 3-kinase (PI3-K) [26]–[28]. Kinases activated downstream in this pathway include PKB/Akt and the atypical protein kinase C iota (PKCι) [29], [30], [31]–[34]. The toxicities of huntingtin and ataxin-1 gene products are modulated by their phosphorylation states [35], [36], but while the role of PKB/Akt activity has been studied in this context nothing is known of the role of PKCι.

As a starting point the current study sought to address the role of MAPK signaling pathways in polyglutamine disorders including Huntington's disease and SCA-1. Our findings suggest that expanded polyglutamine proteins mediate adverse effects through activation of p38MAPK signaling and that this cytotoxicity is antagonized by PKCι, which enhances protective signaling through the ERK pathway. We show that pharmacological inhibition of p38MAPK rescues cells from polyglutamine-induced cell death whereas inhibition of ERK signaling or depletion of PKCι by RNA interference enhances cytotoxicity.

## Methods

### Reagents and antibodies

Custom RNA interference duplexes were synthesized by Dharmacon RNA Technologies Inc. (Lafayette, CO, USA). A control duplex having the following sense RNA sequence AUUCUAUCACUAGCGUGACUU (non-specific control duplex) was purchased from Dharmacon Research, Inc and used as a control. RNA duplex concentrations were determined by measuring absorbance at 260 nm and calculating concentrations using extinction coefficients provided by the manufacturer. Propidium iodide and MTT reagents were purchased from Sigma-Aldrich Canada Ltd. (Oakville, ON, Canada). P38MAPK inhibitors, SKF86002 and SB202190 were purchased from Calbiochem (San Diego, CA, USA) and Biosource (Camarillo, CA, USA) respectively. The MEK inhibitor U0126 was from Promega (Madison, WI, USA). The goat polyclonal antibodies nPKCæ (used to detect PKCι) and ataxin-1 were from Santa Cruz Biotechnology Inc. (Santa Cruz, CA, USA). The mouse monoclonal phospho-p38MAPK and phospho-ERK 1/2 antibodies and the rabbit polyclonal p38MAPK antibody were from Cell Signaling Technology (Beverly, MA, USA). Pan ERK monoclonal antibody was from Transduction Laboratories (Lexington, KY, USA). GFP, Htt-25 and Htt-103 were detected with a mouse monoclonal AFP antibody purchased from Quantum Biotechnologies Inc.(Montréal, Québec, Canada). Phospho-ATF2 and total ATF2 levels were detected with rabbit polyclonal antibodies purchased from Cell Signaling Technology (Beverly, MA, USA). The mouse monoclonal actin antibody was purchased from Sigma-Aldrich Canada (Oakville, ON).

### Expression constructs and transgenic mice

The pEGFP-N1 expression construct which served as a control in transient transfection experiments was purchased from Clontech (Palo Alto, California, USA). The Htt-25 and Htt-103 expression constructs (gifts from Dr. Ron Kopito) contain a synthetic insert encoding exon 1 of human Huntingtin containing a polyglutamine tract of either 25Q or 103Q fused to the yellow fluorescent reporter protein (YFP). The plasmids encoding the full length human ataxin-1 proteins with a polyglutamine tract of 30Q or 83Q were a gift from Dr. Huda Zoghbi. The origin of the B05 transgenic line carrying a mutant Ataxin-1 allele with 82 CAG repeats and the A02 line with a CAG repeat of 30 codons was described in a paper from the laboratory of Dr. Harry Orr [37], from whom these lines were obtained.

### Cell culture and transfections

The human U87MG cell line (a gift from Dr. W. Cavenee, Ludwig Institute for Cancer Research, La Jolla, CA) was maintained at 37°C and 5% CO_2_ in Dulbecco's modified Eagle's medium (DMEM) supplemented with 100 units/ml penicillin, 100 µg/ml streptomycin, 2 mM glutamine and 10% (v/v) of a 2∶1 mixture of donor bovine serum and fetal bovine serum. For RNA interference experiments, cells were transfected using Oligofectamine (Invitrogen Canada, Inc., Burlington, ON) as per the supplier's protocol. Final concentrations of RNA in the transfections were 5.3 nM for siPKCιA and 20 nM for siPKCιB. Control RNA concentrations were matched to the specific siRNA duplex used in the experiment. For transient transfections, cells were plated in either 96- or 6 well dishes 24 hours prior to transfections. Subsequently, they were transfected using GeneJuice Transfection Reagent (Novagen, Madison, WI, USA) as per the supplier's protocol. 0.5 µg of plasmid DNA was used in each well of a 96 well dish. A total amount of 3 µg of plasmid DNA was used in each well of a 6 well dish. For p38MAPK inhibition experiments using SKF86002 and SB202190, cells in 96 well plates were transfected with RNA duplexes. 24 hours post-transfection, cells were pre-treated for 2 h with 20 µM of the respective inhibitor. ERK inhibition experiments were performed in a similar manner using the MEK inhibitor U0126 at a final concentration of 20 µM. Following this incubation period cells were transiently transfected with various expression constructs.

### Survival assays

Survival assays were performed by MTT, trypan blue exclusion and flow cytometry. For MTT assays, cells in 96 well microtitre plates were transfected with RNA duplexes as described above. 6 h post-transfection, they were transiently transfected with the GFP control vector, Htt-25, Htt-103, Atx-30 or Atx-83 as indicated. 24 h post-transfection of the plasmid DNA cell survival was assessed using the MTT (3-(4,5-cimethylthiazol-2-yl)-2,5-diphenyl tetrazolium bromide) assay as described previously [38]. Background values were determined by carrying out the assay in wells containing media without cells. Toxicity was measured by trypan blue exclusion in pooled fractions consisting of attached and detached cells. For flow cytometry experiments, adherent and non-adherent cells were harvested and fixed with 70% (v/v) ethanol in PBS. Cell nuclei were stained with propidium iodide. DNA content was analyzed by flow cytometry using a BD LSR flow cytometer (Becton Dickinson, San Jose, CA). Data was acquired using Cell Quest software (Becton Dickinson, San Jose, CA) and were analyzed using Mod Fit LT software (Verity Software House, Inc., Sopsham, ME).

### Western blot analysis

U87MG cells were harvested in protein lysis buffer consisting of 100 mM Tris pH 6.8, 20 mM DTT, 4% SDS, 5% glycerol. Protein concentrations were determined using the Bradford assay reagents (Bio-Rad, Hercules, CA, USA). Reduced proteins were separated through 4–12% bis-tris polyacrylamide gels using an Xcell II min cell system (Invitrogen, San Diego, CA, USA). Proteins were transferred onto PVDF nylon membranes (Amersham Pharmacia Biotech, Buckinghamshire, UK) and stained with amido black prior to probing with the appropriate primary antibody. Proteins were detected using the HRP method and SuperSignal West Pico Chemiluminescent Substrate reagents (Pierce, Rockford, IL, USA). Proteins were visualized using the GeneGnome (Syngene, Frederick, MD,USA). Sequential probing of membranes was performed after stripping with the use of Western Blot Stripping Buffer (Pierce, Rockford, IL, USA) for 30 min at room temperature. Mouse cerebella were harvested by homogenization in protein lysis buffer (20 mM Tris-HCl pH 7.5, 150 mM NaCl, 0.5 mM EDTA, 1% NP-40 and 20% glycerol) containing the following protease and phosphatase inhibitors: 200 µg/ml phenylmethylsulfonyl fluoride, 5 µg/ml leupeptin, 2 µg/ml aprotinin, 200 µM NaF, 200 µM NaPPi and 10 mM NEM. Soluble protein was quantified as described above. Proteins were resolved on a 10% SDS-polyacrylamide gel and electroblotted onto a Hybond C nitrocellulose membrane (Amersham Biosciences Corp, Baie d'Urfé, QC). The membranes were stained with Ponceau S prior to immunoblotting with the appropriate primary antibody. Proteins were visualized as described above.

### Immunohistochemistry

Cerebella from age-matched nontransgenic, A02 and B05 mice were excised and fixed in 10% phosphate-buffered formalin overnight at room temperature. Tissues were paraffin-embedded and sectioned sagitally using a microtome at a thickness of 5 µm. Deparaffinized sections were heated in a solution of 10 mM sodium citrate (pH 6.0) in 700W microwave for 10 minutes. Endogenous peroxidase activity was blocked by incubating in methanol containing 3% hydrogen peroxide for 20 minutes. Sections were washed with PBS (pH 7.4) and incubated for 30 minutes with 1.5% normal goat serum (Santa Cruz Biotechnologies Inc., SC, CA, USA) to block nonspecific binding. Sections were then incubated overnight at 4°C with the phospho-p38MAPK antibody (Cell Signaling Technology, Beverly, MA, USA). The reaction product was visualized with the ABC system (DAKO Diagnostics Canada Inc.). The use of animals in these experiments followed the guidelines of the Animal Care Committee of the University of Ottawa and was approved under protocol number ME-212.

### Statistical analysis

Unless otherwise indicated, all values are represented as the average of three independent experiments performed in triplicate, with error bars indicating standard error of the mean. Statistical significance was determined by a two tailed Student's t-test. Values were considered significant when P<0.05.

## Results

### PKCι modulates the sensitivity of cells to polyglutamine-induced cellular death

**Figure 1:**
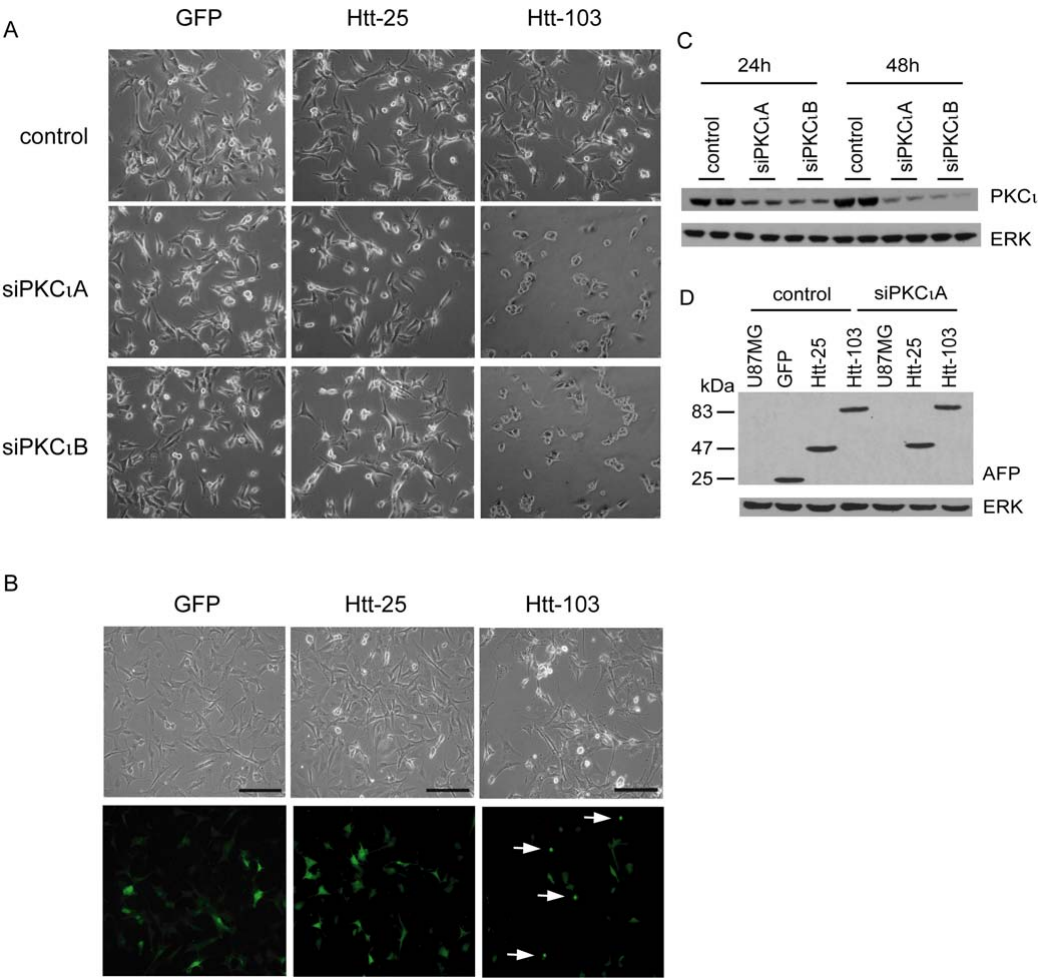
Morphological alterations in PKCι depleted cells expressing Htt-103. A) U87MG cells transfected with the control or with siPKCιA and siPKCιB were transiently transfected with plasmids encoding GFP, Htt-25 or Htt-103 for 24 hours. Cell morphology was assessed by phase contrast microscopy. An increase in the number of shrunken, rounded and detached cells was observed in PKCι depleted cells expressing Htt-103 when compared to control RNA transfected cells or cells expressing GFP or Htt-25. Magnification was 40×. B) U87MG cells expressing GFP, Htt-25 or Htt-103 for 24 hours were visualized under fluorescence (bottom panel) to assess transfection efficiency. Upper panels represent the same field of view visualized under white light. Arrowheads demonstrate nuclear inclusions in Htt-103 expressing cells. Scale bars represent 100 µm. C) Western blot analysis with a PKCι specific antibody of cell extracts from U87MG cells transfected in duplicate with either the control or siPKCιA and siPKCιB showing the reduction in the protein levels of PKCι at 24 and 48 hours post-transfection. The membrane was re-probed with an antibody directed against Pan-ERK which served as a loading control. D) Western blot analysis of cell extracts from cells transfected with either control or siPKCιA expressing GFP, Htt-25 or Htt-103 with an antibody raised against AFP. No significant difference in the protein levels of GFP, Htt-25 and Htt-103 were observed in extracts from control and PKCι transfected cells. The membrane was re-probed with an antibody directed against Pan-ERK which served as a loading control.

**Figure 2:**
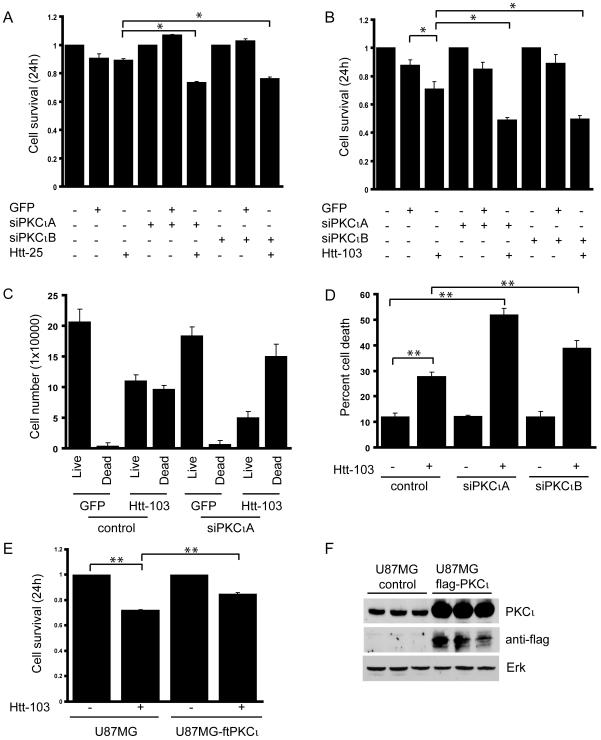
Depletion of PKCι sensitizes cells to polyglutamine induced toxicity. A) and B) U87MG cells transfected for 24 hours with either control or siPKCιA and PKCιB were plated in 96 well dishes. Subsequently, cells were transfected with expression constructs encoding GFP, Htt-25 (A) and Htt-103 (B) and cell survival was measured by MTT assay. A) The survival of mock RNA transfected cells expressing Htt-25 was comparable to cells expressing GFP alone. A slight decrease in cell survival was observed in PKCι depleted cells expressing Htt-25 when compared to GFP transfectants (* p<0.05). B) A marked decrease in cell survival was observed in control RNA transfected cells expressing Htt-103 that was further pronounced in PKCι depleted cells (* p<0.05). C) Survival as measured by trypan blue exclusion of U87MG cells transfected with control or siPKCιA in the presence or absence of Htt-103. In accordance with the MTT assay, an increase in the population of dead cells was observed in control transfected U87MG cells expressing Htt-103 which was further increased in PKCι depleted cells. D) Flow cytometric analysis of cells transfected with either the control or siPKCι expressing Htt-103 showing an increase in the number of dead cells in PKCι depleted cells when compared to control transfected cells. Data represents the average of three independent experiments, with error bars indicating standard deviation (** p<0.01). E) U87MG cells stably expressing flag epitope tagged PKCι (ftPKCι) were transiently transfected with the Htt-103 expression construct for 24 hours. Survival as assessed by MTT analysis revealed a modest increase in survival in PKCι transfectants when compared to the parental U87MG cells (** p<0.01). F) Western blot analysis of triplicate cell extracts from untransfected U87MG and cells stably expressing flag-tagged PKCι with antibodies raised against PKCι and the flag epitope tag respectively. The flag tag specific antibody detected ectopically expressed PKCι in transfected cells, which was absent in the untransfected control cell extracts. The PKCι antibody detected endogenous and exogenous PKCι in lysates from U87MG cells and cells stably expressing PKCι. Pan-ERK served as the loading control. MTT and trypan blue data are represented as the average of three independent experiments performed in triplicate, with errors bars indicating standard error of the mean.

We used a previously described siRNA strategy [38] to investigate the role of PKCι depletion in polyglutamine-induced cytotoxicity. This method specifically depletes PKCι RNA and protein with no effect on other PKC enzymes [38]. We used U87MG cells which have been shown to have an elevated basal ERK activity as a result of increased signaling through the EGFR pathway [39]. We reasoned that if ERK was protective such a cell model would be less sensitive to expanded polyglutamine induced toxicity. The use of cells from a glial as opposed to neuronal lineage is unlikely to be of consequence in that similar results were obtained in glioblastoma and neuroblastoma cell lines (as described below). To assess whether the depletion of PKCi would affect cell survival in the presence of an expanded polyglutamine protein, U87MG cells were transfected with a control or one of two siPKCι RNAs (siPKCιA and siPKCιB). Cells were then transiently transfected with either a GFP control plasmid or constructs encoding exon 1 of the Huntingtin protein containing a normal polyglutamine tract of 25Qs (hereafter referred to as Htt-25) or with a pathogenic tract of 103Qs (hereafter referred to as Htt-103) fused to the yellow fluorescent protein (YFP) reporter. Similar expression constructs encoding exon 1 of the Htt protein with an expanded polyglutamine tract have been previously used in cell culture models of polyglutamine toxicity [6], [40] and in the generation of the well characterized R6/2 transgenic mouse line [41]; R6/2 mice develop a progressive neurological phenotype with motor symptoms resembling those in HD [42]. By phase contrast microscopy, a pronounced effect was observed in PKCι depleted cells expressing Htt-103 wherein a significant increase in the number of shrunken, rounded and detached cells was noted (Figure 1A). Analogous to other cell culture systems used in the study of polyglutamine biology (3T3, PC12, SHY-5Y cells, etc), U87MG cells expressing Htt-103 were found to accumulate visible nuclear inclusions as early as 24 hours post-transfection (Figure 1B). No such inclusions were observed in cells expressing GFP alone or Htt-25 (Figure 1B). Depletion of PKCι was assessed by Western blot analysis with an antibody recognizing PKCι in extracts from U87MG cells transfected with either the control or siPKCιA and siPKCιB; a reduction in the protein levels was observed at 24 and 48 hours post-transfection (Figure 1C). The transfection efficiency of the Htt proteins in U87MG cells was estimated at ∼80% as assessed by fluorescence microscopy (Figure 1B). Similar levels of expression of GFP, Htt-25 and Htt-103 were confirmed by Western blot analysis of extracts from transfected cells with an antibody specific for the fluorescent protein reporter (Figure 1D). Quantification of survival with the use of a metabolic assay (MTT) revealed that the depletion of PKCι sensitized cells to the expression of Htt-103 such that survival was reduced by approximately 25% when compared to cells transfected with the control RNA (Figure 2B). The survival of U87MG cells transfected with an Htt-25 expression plasmid was no different then that of cells expressing GFP alone (Figure 2A). When compared to GFP transfectants, the depletion of PKCι mildly sensitized cells to the expression of Htt-25 but to a lesser extent than did expression of Htt-103 (Figure 2A and B). The data obtained by MTT analysis were consistent with survival as measured by the trypan-blue exclusion method (Figure 2C) and by flow cytometric analysis (Figure 2D) of Htt-103 transfected cells, both of which revealed an increase in cell death in PKCι depleted cells when compared to control RNA transfectants. These data suggested that the depletion of PKCι was sensitizing cells to the expression of expanded polyglutamine proteins. As assessed by MTT, the exogenous expression of flag-tagged PKCι was found to modestly increase the resistance of cells to the toxic effects associated with expression of Htt-103 (Figure 2E). The overexpression of PKCι in these stable transfectants was confirmed by Western blot analysis with both the PKCι and flag tag antibodies respectively (Figure 2F).

### Impaired ERK activation sensitizes cells to polyglutamine-expanded proteins

**Figure 3:**
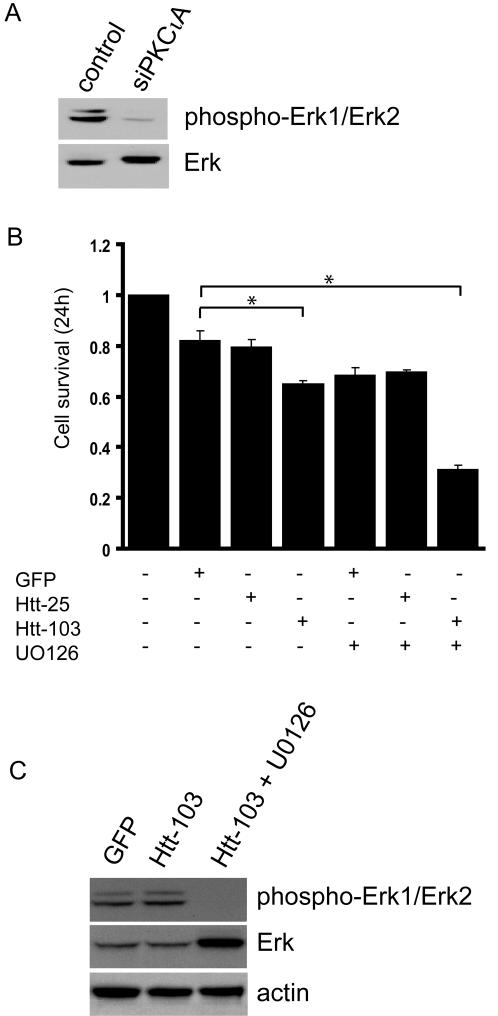
PKCι-mediated ERK activation protects cells from expanded polyglutamine-induced cytotoxicity. A) Western blot analysis of cell extracts from control and siPKCιA transfectants with the phospho-ERK specific antibody. The basal levels of ERK phosphoprotein were significantly reduced in PKCι depleted cells when compared to control RNA transfectants. Total ERK levels were assessed with the pan ERK antibody which also served as a loading control. B) U87MG cells were pre-treated with the MEK inhibitor U0126 for 2 hours prior to transfection with GFP, Htt-25 and Htt-103. Cell survival was assessed by MTT 24 hours post-transfection. Blockade of ERK in Htt-103 expressing cells resulted in a significant reduction in cell survival when compared to untreated Htt-103 expressing cells (* p<0.05). Data are represented as the average of three independent experiments performed in triplicate, with error bars indicating standard error of the mean. C) Western blot analysis of extracts from untreated and U0126 treated U87MG cells expressing Htt-103 with the phospho-ERK antibody confirming the blockade of ERK phosphorylation in U0126 treated cells. Pan-ERK was used to detect total ERK levels and actin served as a loading control.

It has been previously reported that PKCι is positioned upstream of the mitogen-regulated kinase ERK [43] and it was therefore conceivable that PKCι depletion would affect ERK activation. To test this hypothesis, we examined the basal levels of activated ERK in PKCι depleted cells. Cell extracts from control or siPKCιA RNA transfected cells were analyzed by Western blot analysis with an antibody recognizing phospho-ERK. The analysis revealed a reduction in ERK phosphorylation in PKCι depleted cells when compared to the levels in U87MG cells transfected with the control RNA (Figure 3A). These data suggested that U87MG cells have elevated basal levels of activated ERK most probably due to the constitutively active EGFR pathway and that PKCι depletion affects ERK induction. To investigate specifically whether the loss of ERK signaling due to PKCι depletion was the basis for the increased sensitivity observed in PKCι depleted cells to expanded polyglutamine protein expression, we made use of U0126, a specific inhibitor of MEK (positioned directly upstream of ERK). U87MG cells were either untreated or treated with U0126 prior to transfection with the GFP control vector, Htt-25 or Htt-103 plasmids. Twenty-four hours post-transfection, cell survival was assessed by the MTT assay. The data presented in figure 3B revealed that blockade of ERK recapitulated the findings in PKCι depleted cells: the survival of cells expressing Htt-103 was significantly compromised (Figure 3B). The survival of Htt-25 expressing cells treated with the inhibitor was comparable to that of GFP transfectants (Figure 3B). The efficient blockade of ERK activation in U0126 treated cells was confirmed by Western blot analysis of cell extracts from cells transfected with Htt-103 with the phospho-ERK specific antibody (Figure 3C). The data in Figure 3C also revealed that the expression of the expanded polyglutamine protein has no effect on ERK induction when compared to GFP transfectants. Taken together, they suggest that the status of ERK is strictly dependent on PKCι and not the expression of the expanded polyglutamine protein.

### Expression of Htt-103 is associated with induction of p38MAPK and its pharmacological blockade rescues cells from polyglutamine-induced toxicity

**Figure 4:**
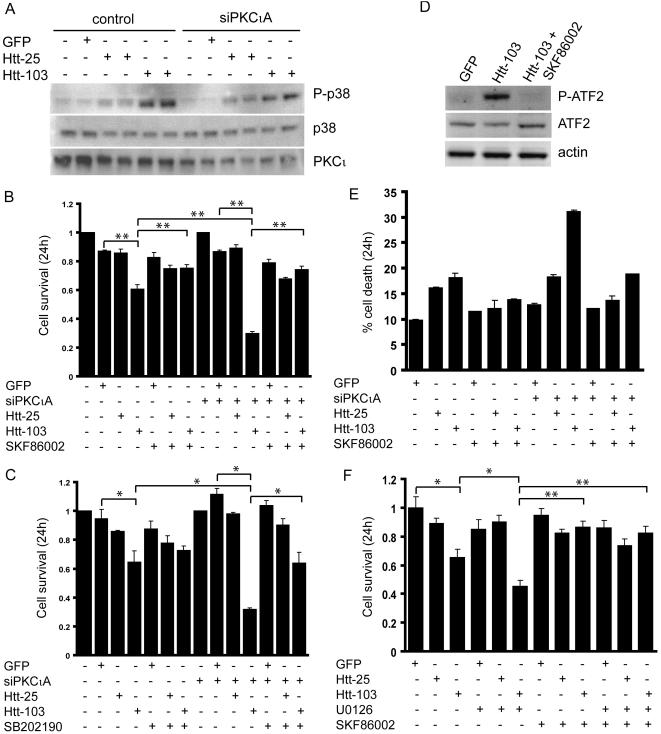
Expanded polyglutamine proteins induce p38MAPK. A) Western blot analysis of extracts from control and siPKCιA transfected cells expressing GFP, Htt-25 or Htt-103 with the phospho-38MAPK antibody showing the phosphorylation of p38MAPK in extracts from control and siPKCιA transfected cells expressing Htt-103. Phospho-p38MAPK levels were slightly increased in extracts from cells transfected with Htt-25 when compared to GFP transfectants. The levels of total p38MAPK remained unchanged in all extracts as assessed by Western blot analysis with the p38MAPK antibody. Efficient depletion of PKCι was confirmed by re-probing the membrane with the PKCι specific antibody. B) and C) Control or siPKCιA transfected cells were either left untreated or were pre-treated with p38MAPK inhibitors, SKF86002 (B) and SB202190 (C) for 2 hours prior to transfection with GFP, Htt-25 or Htt-103. 24 hours post-transfection, cell survival was assessed by MTT which revealed an increase in survival of Htt-103 expressing cells by treatment with SKF86002 in both control and siPKCιA transfected cells (** p<0.01). The survival of Htt-103 expressing cells treated with SB202190 was less pronounced when compared to SKF86002 treated cells but was still statistically increased in PKCι depleted cells when compared to the untreated counterparts (* p<0.05). D) Western blot analysis of cell extracts from cells expressing Htt-103 that were either untreated or treated with SKF86002 with the phospho-ATF2 antibody. The analysis revealed an abrogation of ATF2 phosphorylation in Htt-103 expressing cells treated with SKF86002. Re-probing the membrane with an ATF-2 antibody revealed no significant difference in the total levels of ATF2 protein. Actin served as a loading control. E) Flow cytometric analysis of control and siPKCιA transfected cells expressing Htt-103 that were either left untreated or treated with SKF86002. The survival of SKF86002 treated, Htt-103 expressing cells was significantly improved when compared to the untreated counterparts and was comparable to the survival of GFP and Htt-25 transfectants in both the mock and PKCι depleted cells. Data is represented as the average of three independent experiments performed in duplicate with error bars indicating standard error of the mean. F) U87MG cells transfected with GFP, Htt-25 and Htt-103 were treated with SKF86002 in combination with U0126. Survival was assessed by MTT assay 24 hours post-transfection. As described above, treatment of Htt-103 expressing cells with U0126 resulted in reduced viability (* p<0.05) whereas treatment with SKF86002 alone or in combination with U0126 rescued cells from polyglutamine toxicity to a level that was similar to p38MAPK inhibition alone (** p<0.01). Data are represented as the average of three independent experiments performed in triplicate, with error bars indicating standard error of the mean.

Given that the blockade of ERK signaling preferentially sensitized Htt-103 expressing cells when compared to Htt-25 transfectants, we reasoned that the expanded polyglutamine may be affecting stress-inducible pro-apoptotic pathways. The activation of the p38MAPK pathway in response to environmental and genotoxic stress is well characterized [44]–[46] and its induction in response to amyloid beta treatment has been well documented [47], [48]. Expanded polyglutamine proteins have recently been shown to induce death in cell culture models (reviewed in [49]) but the role of p38MAPK has not been investigated. To investigate the role of this kinase, we analyzed cell extracts from control and PKCι depleted cells expressing GFP, Htt-25 and Htt-103 by Western blot analysis with a phospho-p38MAPK antibody. The analysis revealed that the expression of Htt-103 resulted in a similar increase in p38MAPK phosphorylation in both the control and siPKCιA transfected cells. These data suggested that the status of PKCι has no effect on expanded polyglutamine induced p38MAPK activation (Figure 4A) and that the increased sensitivity observed in PKCι depleted cells was a reflection of a diminished activation of ERK. The levels of phospho-p38MAPK remained unchanged in GFP expressing cells and were minimally affected in Htt-25 transfectants (Figure 4A). This suggested that the activation of p38MAPK may be the basis for the increased cell death observed in Htt-103 expressing cells and that interfering with its phosphorylation may rescue cells from polyglutamine-induced toxicity. Inhibition of p38MAPK with the use of SKF86002, a specific p38MAPK inhibitor, resulted in a significant rescue of Htt-103 expressing U87MG control and siPKCιA transfected cells such that their survival was comparable to Htt-25 and GFP transfectants treated with the inhibitor (Figure 4B). Similar results were obtained by blockade of p38MAPK with the use of SB202190, a different p38MAPK inhibitor; its inhibition resulted in a statistically significant increase in cell survival of PKCι depleted cells expressing Htt-103 (Figure 4C). The efficient blockade of p38MAPK activation in SKF86002 treated cells was confirmed by Western analysis of cell extracts from Htt-103 expressing cells with a phospho-ATF2 antibody, a downstream target of p38MAPK (Figure 4D). Flow cytometric analysis of Htt-103 expressing cells treated with SKF86002 revealed that inhibiting p38MAPK increased the survival of Htt-103 transfectants in both control and PKCι depleted cells (Figure 4E). To further dissect the relative importance of ERK and p38MAPK in polyglutamine-induced death, we treated GFP, Htt-25 and Htt-103 expressing U87MG cells with SKF86002 in combination with U0126. By MTT analysis, we found that pharmacological inhibition of p38MAPK alone or in combination with ERK inhibition resulted in a similar and significant rescue of cells from death associated with expression of Htt-103 (Figure 4F). These data suggest that the induction of p38MAPK contributes to polyglutamine-induced cytotoxicity and that whether in the presence or absence of activated ERK, its inhibition is sufficient to block cell death.

### Full-length expanded human Ataxin-1 protein induces cell toxicity in a p38MAPK dependent manner

**Figure 5:**
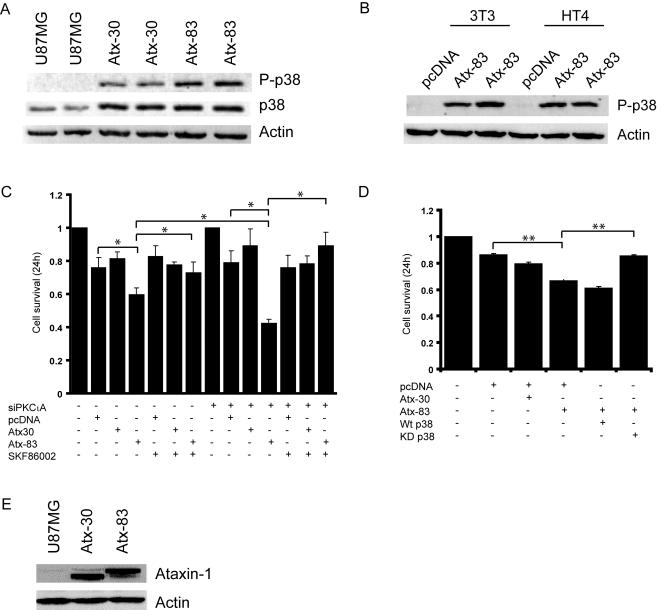
Expanded ataxin-1 toxicity is mediated through induction of p38MAPK. A) Western blot analysis of duplicate cell extracts from U87MG cells expressing Atx-30 and Atx-83 with the phospho-p38MAPK antibody. An increase in phosphorylated p38MAPK was observed in lysates from Atx-83 expressing cells when compared to lysates from mock transfected or cells expressing Atx-30. Total levels of p38MAPK were increased in lysates from cells transfected with either Atx-30 and Atx-83 when compared to mock transfected cells as assessed by re-probing of the membrane with the p38MAPK antibody. Actin served as a loading control. B) HT4 and NIH-3T3 cells were transiently transfected in duplicate with the Atx-83 expression construct. Cell extracts were analyzed by western blot analysis with the phospho-p38MAPK antibody. p38MAPK activation was observed in cell extracts from both NIH-3T3 and HT4 cells expressing Atx-83. The induction of p38MAPK was not observed in lysates from cells expressing an empty vector control. C) Untreated or SKF86002 treated control or siPKCιA transfected cells were transfected with Atx-30 or Atx-83 for 24 hours and cell survival was assessed by MTT. The analysis revealed a decrease in survival of Atx-83 expressing cells in control RNA transfected cells that was significantly more pronounced by PKCι depletion. Blockade of p38MAPK increased survival of Atx-83 expressing cells in both control and siPKCι transfectants such that it was comparable to the survival of Atx-30 expressing cells. Data represent the average of three independent experiments performed in triplicate, with error bars indicating standard error of the mean (* p<0.05). D) U87MG cells were co-transfected with Htt-103 and either empty vector alone or expression constructs encoding wild-type (wt p38) or dominant-negative (KD) p38MAPK alpha. 24 hours post-transfection, cells were analyzed by MTT which revealed a statistically significant increase in survival in cells co-expressing Htt-103 and dominant-negative p38MAPK alpha (** p<0.01). The survival of cells expressing empty vector control alone or co-expressing Htt-25 with empty vector was not significantly different. E) Western blot analysis of extracts from Atx-30 and Atx-83 transfected cells with an ataxin-1 specific antibody revealing a similar level of expression. Actin served as a loading control.

To investigate whether the depletion of PKCι and p38MAPK pathways represent a general mechanism of expanded polyglutamine toxicity, we transfected control or PKCι depleted cells with an expression construct encoding the full length ataxin-1 gene product with an expanded polyglutamine tract of 83Q (hereafter referred to as Atx-83). The length of the polyglutamine repeat in normal, unaffected humans is from 6 to 40 residues and mice expressing full length ataxin-1 with 30Qs (Atx-30) show no phenotype effects [37]; the Atx-30 expression was therefore a suitable control for the expanded (83Q) protein in our experiments. We were unable to detect expression of an ataxin-1 protein with only 2 glutamine residues and speculate that this variant may be unstable (data not shown). Western blot analysis of cell extracts from Atx-30 and Atx-83 transfected cells with the phospho-p38MAPK antibody revealed an increase in p38MAPK activation in Atx-83 expressing cells when compared to Atx-30 and parental U87MG cells (Figure 5A). Additionally, the ectopic expression of Atx-30 and Atx-83 resulted in an increase in total levels of p38MAPK as assessed by Western analysis of the same membrane with the antibody raised against total p38MAPK (Figure 5A). An increase in p38MAPK activation in response to ectopic expression of Atx-83 was also observed in NIH-3T3 fibroblasts and HT4 neuroblastoma cells suggesting that its induction represents a cell type independent mechanism of polyglutamine cytotoxicity (Figure 5B). By MTT assay, we found that the survival of control RNA transfected U87MG cells expressing Atx-83 was reduced when compared to cells expressing the non-expanded Atx-30 counterpart 24 hours post-transfection (Figure 5C). The sensitivity of cells expressing Atx-83 was significantly increased in PKCι depleted cells; survival was reduced by approximately 20% when compared to control RNA-transfected cells expressing an empty vector control (Figure 5C). Pharmacological inhibition of p38MAPK with the use of SKF86002 in Atx-83 expressing cells recapitulated the findings in Htt-103 transfectants; a statistically significant increase in cell survival was observed in control RNA transfectants and was more pronounced in PKCι depleted cells (Figure 5C). To confirm that the rescue observed in SKF86002 treated cells was attributable to blockade of p38MAPK signaling, we transiently co-transfected U87MG cells with the Atx-30 or Atx-83 plasmids in conjunction with constructs encoding either flag tagged wild-type p38 alpha (wt p38) or its dominant-negative kinase dead counterpart (KD p38). These expression constructs have previously been used to examine the contribution of p38MAPK signaling in cultured cells [50], [51]. By MTT analysis we found that expression of the kinase dead p38MAPK increased survival of Htt-103 expressing cells in a similar manner to blockade with SKF86002 suggesting that the decrease in survival is due to activation of p38MAPK. The expression of wt p38 had no significant impact on survival of Atx-83 expressing cells (Figure 5D). The expression levels of Atx-30 and Atx-83 were similar as assessed by Western blot analysis with an ataxin-1 specific antibody (Figure 5E).

### Expanded polyglutamine protein induced p38MAPK in the cerebella of SCA-1 transgenic mice

**Figure 6:**
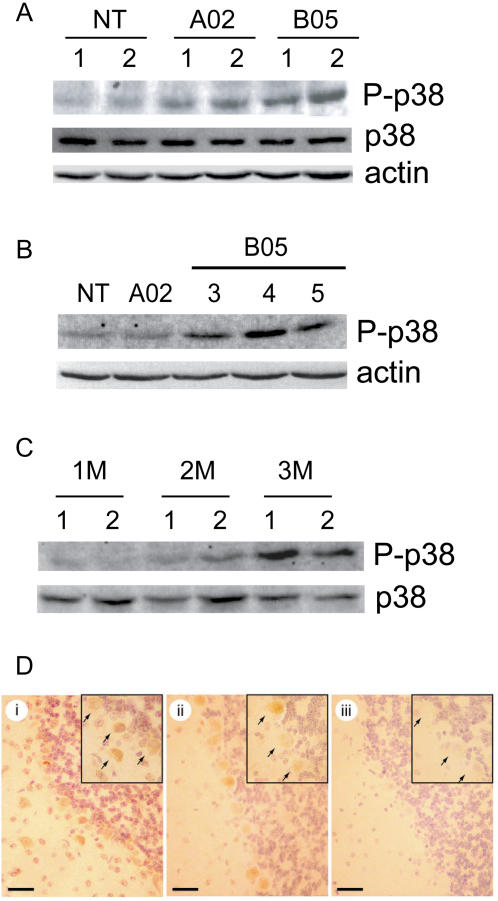
In vivo induction of p38MAPK in SCA-1 mice. A) and B) Western blot analysis of cerebellar extracts from 3 month-old nontransgenic, A02 and B05 mice with the phospho-p38MAPK antibody. An increase in the protein levels of phospho-p38 was detected in the extracts from B05 mice when compared to A02 and nontransgenic control extracts. Total p38MAPK levels were similar as assessed by re-probing the membrane with the p38MAPK antibody or actin. C) Western blot analysis of cerebellar extracts from B05 mice at 1,2 and 3 months of age with the phospho-p38MAPK antibody. The analysis revealed a detectable induction in p38MAPK activation in lysates from mice at 3 month of age. Total levels of p38MAPK were assayed by re-probing the membrane with the p38MAPK antibody which also served as a loading control. D) Immunohistochemistry of mouse cerebella with the phospho-p38MAPK antibody. Panel i) cerebellum of a B05 animal and panel ii) cerebellum of a nontransgenic animal. Immunoreactivity was observed in the cytoplasm and nucleus of Purkinje neurons in B05 and nontransgenic mice. Panel iii) section from a B05 animal stained with the secondary antibody alone demonstrating the absence of immunoreactivity by omission of the primary antibody. Scale bars represent 25 µm.

The *in vivo* induction of p38MAPK was examined in the previously characterized B05 mouse model of spinocerebellar ataxin-1 (SCA-1). In this model a human ataxin-1 cDNA with an expanded CAG tract encoding 82 glutamines is specifically expressed in Purkinje neurons (reviewed in [52]). The A02 transgenic strain expressing a similar construct with a non-pathological expansion of 30 glutamines served as a control. Western blot analysis of cerebellar extracts from aged-matched 3 month old mice with the phospho-p38MAPK antibody revealed phosphorylation of p38MAPK in extracts from nine B05 mice (five of which are shown in Figures 6A and B). In agreement with the findings in cultured cells, the phosphorylation of p38MAPK in lysates from A02 mice was lower than that detected in B05 extracts but slightly increased when compared to lysates from nontransgenic controls (Figure 6A). Contrary to what was observed in lysates from U87MG cells transfected with Atx-30 and Atx-83, re-probing the membrane with the antibody raised against total p38 revealed that total p38MAPK levels remained unchanged in A02 and B05 lysates when compared to nontransgenic control lysates (Figure 6A). We speculate that the induction of total p38MAPK levels may simply represent a response of cultured cells to the expression of Atx-30 and Atx-83. In B05 mice we observed a significant induction in p38MAPK phosphorylation at 3 months of age, while mice at 1 and 2 months of age show little or no detectable p38MAPK phosphorylation (Figure 6C). This activation correlates well with the onset of behavioral and anatomical anomalies in the mouse model of SCA-1. We examined the localization of phosphorylated p38MAPK by immunohistochemistry in cerebella of 3 month old nontransgenic and B05 mice. We found that phosphorylated p38MAPK was primarily localized to the cytoplasm and nucleus of Purkinje neurons (Figure 6D), showing that the increase in the levels of activated p38MAPK (as detected by Western analysis) could be attributed to expanded polyglutamine expression in those cells.

## Discussion

Clear evidence for the essential role of protein kinase C family members in neuronal homeostasis has been provided by neurodegeneration attributable to a loss of function mutation in the *PKCγ* gene in spinocerebellar ataxia type 14 (SCA-14, [53]). No such genetic disorder has been mapped to the *PKCι* gene, but evidence from overexpression studies indicates that PKCι can be protective against a variety of cytotoxic insults including UV damage and chemotherapy [38], [54] and neurotoxic insults including beta amyloid [55]. Conversely, inhibition of PKCι and the closely related PKCζ by the prostate apoptosis-response 4 (PAR-4) protein has been recently shown to increase proteolytic processing of amyloid precursor protein [56], [57] and to exacerbate Aβ accumulation and toxicity in mouse models of Alzheimer's disease [58], [59] suggesting a role for PKCι in modulating survival.

**Figure 7:**
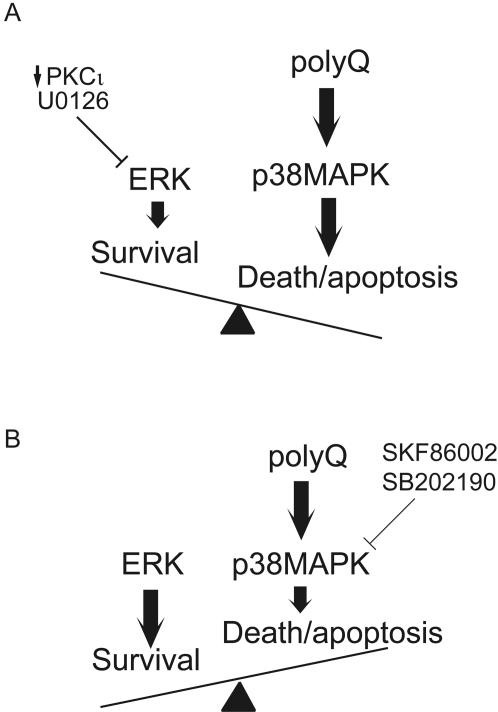
Model of polyglutamine induced toxicity. Activation p38MAPK signaling is counteracted by PKCι-mediated ERK activation in expanded polyglutamine expressing cells. The pharmacological blockade of ERK with U0126 or by PKCι depletion sensitizes cells to polyglutamine-induced death through a mechanism of exaggerated induction of p38MAPK (A). In contrast, inhibition of p38MAPK phosphorylation by SKF86002 or SB202190 rescues cells from polyglutamine toxicity (B). The blockade of both signaling pathways in cells expressing expanded polyglutamine proteins recapitulates blockade of p38MAPK (B) indicating a causative association between p38MAPK induction and polyglutamine induced death.

Using specific MAP kinase inhibitors we have established that p38MAPK is activated in expanded polyglutamine expressing cells and that PKCι-mediated ERK activation can antagonize polyglutamine-induced cell death in a cell culture model. Our data are in accordance with a recent report demonstrating the protective effects of ERK activation in expanded polyglutamine expressing cells [19]. Based on our findings, we propose a mechanism (schematically depicted in Figure 7) wherein p38MAPK induction contributes significantly to the toxicity observed in expanded polyglutamine expressing cells while ERK activation serves to counteract its effects. The fate of cells expressing polyglutamine proteins would therefore seem to be determined, in part, by comparing the activation state of the two signaling cascades. In this model the ERK cascade would generate a pro-survival signal in response to PKCι-mediated input. The p38MAPK cascade would generate a pro-death output specifically in response to the expanded polyglutamine protein. If the p38MAPK signal outweighed the ERK signal (as is the case by expression of expanded polyglutamine proteins or by blockade of ERK and/or PKCι signaling) the cell would respond by activating its cell death program. In the presence of expanded polyglutamine proteins the simultaneous blockade of both ERK and p38MAPK signaling pathways was found to be functionally equivalent to blockade of p38MAPK alone suggesting that the inhibition of p38MAPK was sufficient to block cell death regardless of the presence or absence of activated ERK (Figure 4F). To promote the survival of neurons in neurodegenerative disorders it may therefore suffice to block p38MAPK signaling (there may be no added therapeutic benefit in promoting the ERK-mediated survival signal, despite previously published evidence that ERK activation promotes survival of polyQ-expressing PC12 cells [19]). Consistent with our supposition that p38MAPK blockade should be the therapeutic objective is recent evidence demonstrating that the promotion of ERK-mediated signaling may ultimately compromise neuronal viability ([60]–[62] and reviewed in [63], [64]).

A recent report has implicated activated stress inducible JNK in a cell culture model of HD [19] and its pharmacological blockade resulted in a statistically significant but partial inhibition of cell death [19]. Our data do not allow us to formally exclude a role for JNK, and it is conceivable that the concerted action of both these pathways mediate adverse effects on polyglutamine expressing cells. Whether or not this is the case the almost complete rescue of cell death by inhibition of p38MAPK under our experimental design suggests a significant contribution of this kinase in mediating toxicity.

The model presented in Figure 7 is based on data from polyglutamine tracts in two quite different contexts (an expanded polyglutamine tract appended to exon 1 of the huntingtin protein and the pathogenic form of full length ataxin-1), suggesting that it may have applicability to expanded polyglutamine proteins in general. The activation of p38MAPK was detected in cultured mammalian cells of different origins (glioblastoma, fibroblasts and cells of neural lineage) and more importantly in cerebellar Purkinje neurons of transgenic mice expressing the neuropathogenic ataxin-1 cDNA at the age of onset of pathology (Figure 6 and [65]). In conjunction with recent reports demonstrating p38MAPK induction in cellular [66] and animal models of Alzheimer's disease [67], [68] and amyotrophic lateral sclerosis [69]–[71], our data suggest that blockade of p38MAPK may have broad utility in delaying the progress of neurodegenerative diseases, even those that do not involve expanded polyglutamine proteins. Consistent with this supposition is the finding that inhibition of p38MAPK is beneficial in mouse models of disease [72], [73] and in the suppression of human inflammatory conditions [74]–[76]. Here we demonstrate that pharmacological blockade of p38MAPK may potentially be an efficacious intervention for the polyglutamine disorders. Such an intervention may not only attenuate regional inflammation (reviewed in [77]) and decrease the phosphorylation of HSP27 (a downstream target of p38/MAPKAP 2/3 whose phosphorylation status has been shown to modulate the cytotoxity of polyglutamine expressing cells, [78]), but may delay or preclude the otherwise inexorable neuronal loss that is associated with these diseases.

Other therapeutic modalities may become apparent as the events downstream of p38 MAPK activation by polyglutamine proteins become known. At this point it is not at all clear how many and which of the several pathogenic mechanisms might be affected by p38MAPK signaling. One plausible scenario is that p38MAPK activation leads to transcriptional dysregulation through negative effects on pivotal transcriptional regulators. For example, the levels of the p300/CBP histone acetyltransferase enzymes are known to be affected by expanded polyglutamine proteins [6], [79]–[81], and their loss correlates with reduced expression of a set of target genes whose importance to neuronal homeostasis is well established [79], [82]. It has recently been shown that p300 is degraded by the proteasome in response to p38MAPK activation [83] and that partial inhibition of proteolysis may delay the loss of p300/CBP in the SCA-1 model [84]. Consistent with this model, inhibitors of histone deacetylases (HDACs) have been shown to have beneficial effects in counteracting polyglutamine protein toxicity [85]–[88] and recently reviewed in [89]. The histone acetyltransferases would therefore seem promising as downstream targets of p38MAPK, and we are currently seeking a deeper understanding of this relationship.
